# Genetic variants associated with Fabry disease progression despite enzyme replacement therapy

**DOI:** 10.18632/oncotarget.22505

**Published:** 2017-11-18

**Authors:** Francesca Scionti, Maria Teresa Di Martino, Simona Sestito, Angela Nicoletti, Francesca Falvo, Katia Roppa, Mariamena Arbitrio, Pietro Hiram Guzzi, Giuseppe Agapito, Antonio Pisani, Eleonora Riccio, Daniela Concolino, Licia Pensabene

**Affiliations:** ^1^ Department of Experimental and Clinical Medicine, Magna Graecia University, Salvatore Venuta University Campus, Catanzaro, Italy; ^2^ Department of Medical and Surgical Sciences Pediatric Unit, Magna Graecia University, Catanzaro, Italy; ^3^ ISN-CNR, Roccelletta di Borgia, Catanzaro, Italy; ^4^ Department of Medical and Surgical Sciences, Magna Graecia University, Catanzaro, Italy; ^5^ Department of Nephrology, University Federico II, Naples, Italy

**Keywords:** Fabry disease, enzyme replacement therapy, DMET, ADH genes, oxidative stress

## Abstract

Enzyme replacement therapy (ERT) has been widely used for the treatment of Fabry disease, a rare X-linked recessive disorder due to absent or reduced activity of lysosomal enzyme α-galactosidase A. It is still unclear why some patients under ERT show disease progression typically with renal, cardiovascular and cerebrovascular dysfunctions. Here, we investigated the involvement of drug absorption, distribution, metabolism, and excretion gene variants in response variability to ERT, genotyping 37 patients with the Affymetrix Drug Metabolizing Enzyme and Transporters (DMET) Plus microarray. We found three single nucleotide polymorphisms in human alcohol dehydrogenase (ADH)4 gene (rs1126670, rs1126671, rs2032349) and one in ADH5 gene (rs2602836) associated with disease progression (*p* < 0.05). Our data provide a basic tool for identification of patient with ERT non-response risk that may represent a framework for personalized treatment of this rare disease.

## INTRODUCTION

Fabry disease (FD, OMIM #301500) is a rare X-linked recessive disorder characterized by the absence or reduced activity of α-galactosidase A (α-GalA). This enzyme deficiency leads to deposition of globotriaosylceramide (Gb3) in body fluids and in the vascular endothelium of many organs [1]. The initial signs and symptoms appear in childhood or adolescence and include angiokeratoma, acroparasthesia, corneal opacities, hypohidrosis and gastrointestinal symptoms [2–5].

Vascular dysfunction is the main manifestation of later disease progression observed in FD patients, who typically manifest abnormalities of renal function, cardiac defects and cerebrovascular complications, resulting in early demise, typically in the fourth or fifth decade of life [6]. Enzyme replacement therapy (ERT) with recombinant α-GalA has been widely used for the treatment of FD patients. Clinical trials using agalsidase alfa (Replagal^®^ Shire HGT) and agalsidase beta (Fabrazyme^®^ Genzyme Corp) have shown that ERT is safe and well tolerated and is able to remove Gb3 inclusions from smooth muscle, epithelial cells, myocardium and kidney [7–12].

However, it has become evident that the removal of stored Gb3 from endothelial cells does not prevent progression of vascular disease in all patients [13], specifically in advanced stage with renal impairment, suggesting a limited success in treatment. Involvement of modulators in the vascular pathophysiology of FD, unrelated to α-GalA and Gb3 accumulation, such as genetic and environmental factors, has been largely investigated. Several studies reported the association of single nucleotide polymorphisms (SNPs) or mutations in inflammatory and coagulation factor genes, such as interleukin 6 (c.−174G>C), endothelial nitric oxide synthase (p.Glu298Asp), the factor V (p.Arg506Gln), and the gene encoding the vitamin-K-dependent protein Z (c.−13A>G, IVS6 + 79G>A), with an increased risk of cerebral lesions and stroke in patients with FD [14–16]. In the present study, we investigated whether, in addition to α-GalA, genetic variants in genes encoding drug absorption, distribution, metabolism, and excretion (ADME) proteins exert some effect on response variability to ERT in a group of 37 FD patients. We compared the genetic profiling of 1936 variants across 231 genes in 28 responders *versus* 9 non-responders using the Affymetrix Drug Metabolizing Enzyme and Transporters (DMET) Plus platform.

## RESULTS

### Response to ERT

**Figure 1:**
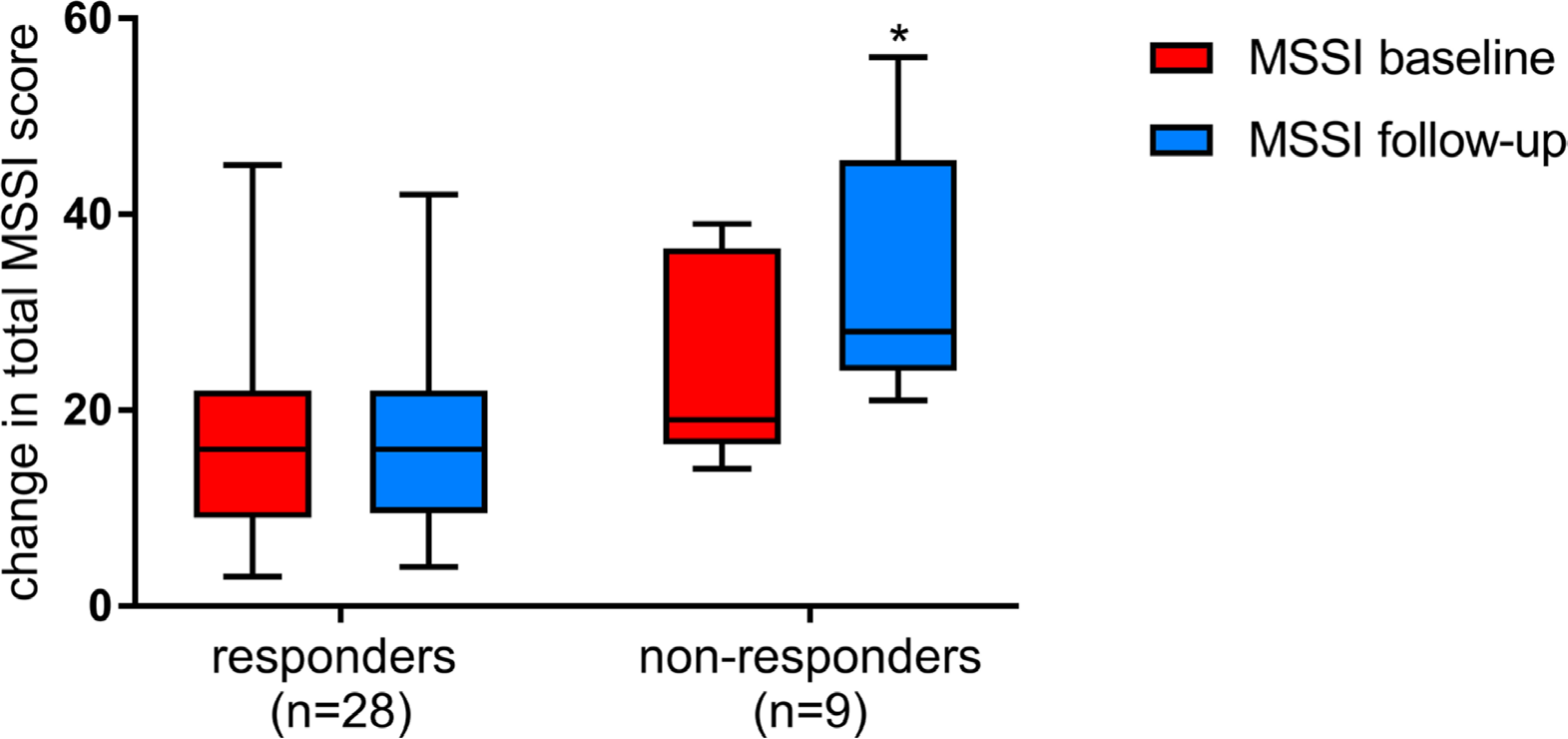
Changes in total MSSI scores among responders and non-responders at baseline (red) and after at least 1 years of ERT (blue) The box plots show, median (rule), interquartile range (box), and minimum and maximum values (whiskers) (GraphPad Prism v.7). ^*^p ≤ 0.05.

**Figure 2:**
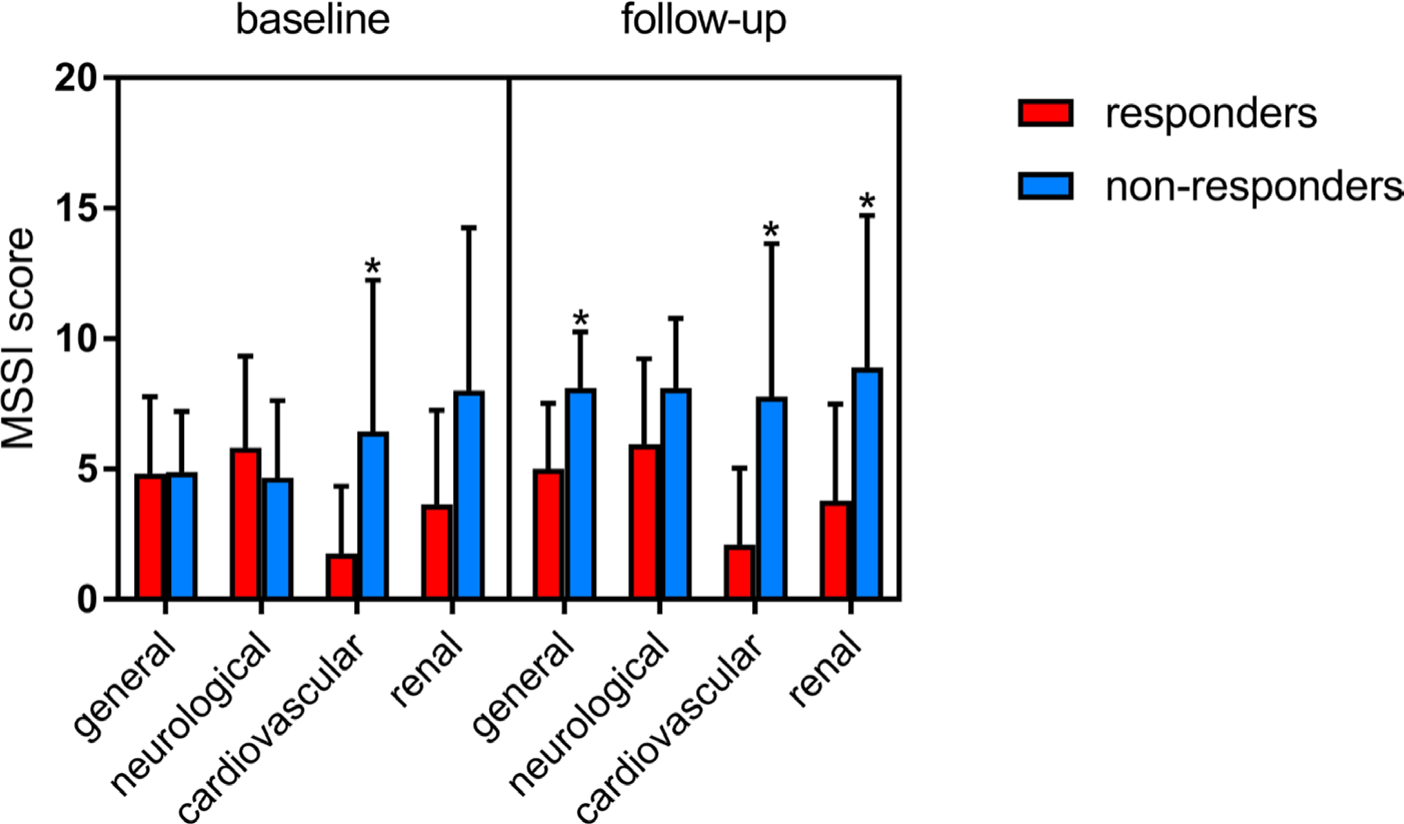
Changes of clinical parameters included in the MSSI score (general, neurological, cardiovascular, renal) among responders and non-responders at baseline and after at least 1 years of ERT (GraphPad Prism v.7) ^*^p ≤ 0.05.

According to Mains Severity Score Index (MSSI) twenty-eight patients were classified as responders and nine as non-responders. At baseline total MSSI score in responders ranged from 3 to 45 (median 24). Twenty-one, six and one patients showed mild, moderate and severe involvement respectively. In non-responders total MSSI score ranged from 14 to 36 (median 25). Six non-responders showed mild while three moderate involvement. Figure 1 shows significant changes in total MSSI score between responders and non-responders after at least 1 years of ERT (median duration of treatment 5 years, range 1–10 years) (*p* = 0.0003). Five responders showed an improvement in total MSSI score (median change −1.8, ranged from −1 to −3), twelve worsened (median change +2.8, ranged from +1 to +8), and ten remained stable. In non-responders the total MSSI score increased with a median change of 9.5, ranged from +4 to +27. When we evaluated MSSI score for single clinical parameters (general, cardiovascular, neurological and renal), we observed a significant difference between the two groups in cardiovascular baseline MSSI score (*p* = 0.001), also after Bonferroni correction (*p* = 0.006). At follow-up non-responders showed an increase in MSSI score for general (*p* = 0.002) and renal (*p* = 0.004) parameters in addition to cardiovascular (*p* = 0.0004) (Figure 2). No correlation has been observed between response to ERT and age, sex and treatment duration.

### Genetic and statistical results

All 37 patients passed QC metrics and produced useable genotypes with an average call rate >95%. Among the 1936 SNPs included in the DMET assay, we used genotyping data from 993 polymorphic SNPs for statistical analysis. The rs953062 in *CYP39A1* failed to meet Hardy-Weinberg equilibrium (HWE) and thus was excluded from further analysis. Three SNPs in *ADH4* gene (rs1126670, rs1126671, rs2032349) and one in *ADH5* (rs2602836) resulted significantly associated with response to ERT (Table 1). The heterozygous genotypes GT (rs1126670), AG (rs1126671) and CT (rs2602836) resulted more frequent in non-responders compared with responders, while the homozygous genotypes CC (rs2032349 and rs2602836) were more frequent in responders. All related genotypes were confirmed with TaqMan SNP genotyping assays.

**Table 1:** Genotypic distribution of SNPs in ADH4 and ADH5 among responders and non-responders

SNP ID^a^	Gene	Chr	Alleles	Genotype(no.)		*P*-value	OR (95% C.I.)
				Responders (*N* = 28)	Non-responders (*N* = 9)		
rs2602836	*ADH5*	4	C/T^*^	CC=15	CC=0	0.0052	0.046 (0.0024 to 0.8636)
				CT=9	CT=8	0.0052	16.89 (1.8251 to 156.2884)
				TT=4	TT=1		
rs1126670	*ADH4*	4	G/T^*^	GG=3	GG=0	0.0002	45.82 (2.3885 to 879.1319
				GT=8	GT=9	0.0015	0.035 (0.0018 to 0.6540)
				TT=17	TT=0		
rs1126671	*ADH4*	4	A/G^*^	AA=4	AA=0	0.0001	54.47 (2.8144 to 1054.0945)
				AG=7	AG=9	0.0015	0.035 (0.0018 to 0.6540)
				GG=17	GG=0		
rs2032349	*ADH4*	4	C/T^*^	CC=27	CC=6		
				CT=1	CT=3	0.0375	0.074 (0.0065 to 0.8414)
				TT=0	TT=0		

SNPs were tested for association using Fisher's exact test. SNPs and genes indicated were limited to those in which *p* < 0.05. SNP ID^*^ is the SNP identifier based on NCBI dbSNP. ^*^genotypes reported as reverse strand.

## DISCUSSION

ERT has been widely used for the treatment of FD. However, it is still unclear why some patients under ERT progress to renal, cardiovascular and cerebrovascular dysfunctions. Growing evidence is supporting the notion that Gb3 acts as a second messenger inducing oxidative stress and inflammation in FD vasculopathy [17–19]. In this context, Biancini *et al.* [20] found decreased levels of antioxidant defenses in FD patients while compared to controls: reduced glutathione and glutathione peroxidase activity and increased superoxide dismutase/catalase ratio in erythrocytes. Also, authors reported increased plasma levels of malondialdehyde and protein carbonyl groups in FD patients, as a consequence of higher lipid peroxidation and protein damage, compared to controls. However, the underlined molecular mechanisms that lead to cell and tissue damage in FD vasculopathy can be only partially explained by Gb3 accumulation, as demonstrating by lack of ERT responsiveness in FD patients despite Gb3 removal.

In this study, we investigated the impact of ADME gene variants on treatment failure in a cohort of 37 FD patients under ERT. We found three SNPs in *ADH4* gene (rs1126670, rs1126671, rs2032349) and one in *ADH5* gene (rs2602836) associated with disease progression. Both genes belong to the human alcohol dehydrogenase (*ADH*) family clustered on chromosome 4q22-23 [21]. Active ADH enzyme is formed by a dimerization interaction between two of nine possible different subunits, each encoded by a unique gene. The *ADH4* gene encodes the human π subunit and contributes to the metabolization of a wide variety of substrates, including ethanol, retinol, other aliphatic alcohols, hydroxysteroids, and lipid peroxidation products. This gene is expressed primarily in the liver and at lower levels in the gastrointestinal tract and spleen. The *ADH5* gene encodes the χ subunit and is involved in the metabolism of alcohols and aldehydes [22]. Unlike other members of the ADH family, *ADH5* is ubiquitously expressed. The *ADH4* rs1126671 at exon 7 results in the amino acid substitutions Val-Ile and might affects the function of the π subunit. The rs1126670 at exon 6 and the rs2032349 at exon 3 are synonymous changes, respectively of Pro-Pro and Ser-Ser. The rs2602836 in *ADH5* gene is located at intergenic level and could be important for gene expression level. In previous study, *ADH* variants have been implicated in the risk for alcohol and drug dependence [23], schizophrenia and autism [24], cancer [25, 26]. *ADH* genes are also involved in the metabolism of 4-hydroxynonenal (4-HNE) to produce alcohol 1,4-dihydroxy-2-nonene (DHN). 4-HNE is an aldehyde which can be formed as secondary product during lipid peroxidation and its levels increase significantly in plasma and tissues in disease associated with oxidative stress, such as atherosclerosis and diabetes, and neurological disorders [27]. Studies have found abundance of 4-HNE in the vascular endothelial and smooth muscle cells. Levels of 4-HNE in vasculature are not only dependent on the rate of lipid peroxidation and 4-HNE synthesis, but also on the removal of 4-HNE adducts by phase II metabolic pathways. Because at high level 4-HNE can react with protein and DNA to form adducts, with consequent toxicity, it is evident that mutations and/or functional SNPs in genes involved in 4-HNE metabolism, such as *ADH* genes, could reduce the rapid intracellular metabolism of this compound and could be crucial for cell survival in a compromised oxidative stress system. ADH4 is highly active in the reduction of 4-HNE, supporting a defense role for cells [28].

We suggest that SNPs in *ADH4/ADH5* genes, observed in this study, could be considered a risk factor, linked to oxidative stress state, for disease progression in FD patients despite enzyme replacement therapy. However, for the absence of functional analysis and the small size of population our study should be considered a hypothesis generating study. Validation of our results has been planned in a larger and independent cohort to evaluate the correlation between 4-HNE plasmatic levels, *ADH4/ADH5* genotypes and MSSI score index. In conclusion, our results provide a basic tool and framework in the light of personalized medicine for FD.

## MATERIALS AND METHODS

### Patients

From January 2004 to May 2014, 37 patients with a clinical and molecular diagnosis of FD have been regularly monitored at the Pediatric Unit of *Magna Graecia* University of Catanzaro and at the Department of Nephrology of *Federico II* University of Naples. Of these, 33 patients received infusions of agalsidase alfa 0.2 mg/kg every other week, the remaining 4 were treated with agalsidase beta every 2 weeks at a dose of 1 mg/ kg. ERT with algasidase alfa was initiated 1–6 years (median:2.3) after diagnosis (median age 35.7 years, range 15–62 years), while ERT with algasidase beta 1–4 years (median: 3.2 years) after diagnosis (median age 41.7 years, range 25–57 years).

Clinical characteristics of patients are summarized in Table 2. Study protocols were approved by institutional ethics committee and written informed consent was obtained from all participants.

**Table 2:** Patient characteristics

	Total	Male	Female
**Total patients (no.)**	37	17	20
**Age (y; median SD)**	40.5 ± 13.3	40.1 ± 16.8	40.9 ± 7.6
**ERT**			
**with Agalsidase alfa**	33	17	16
**with Agalsidase beta**	4	0	4

The characteristics of 37 patients with a clinical and molecular diagnosis of FD enrolled in the study are indicated. Patients have been monitored regularly at the Pediatric Unit of Magna Graecia University of Catanzaro and at the Department of Nephrology of Federico II University of Naples and received ERT with algasidase alfa or beta.

### Treatment outcome

Disease progression and treatment effects in individual patients were assessed longitudinally as changes from baseline using MSSI [29]. The MSSI scoring system consists of four sections that include general, neurological, cardiovascular and renal signs and symptoms [30]. For each component, a single rating was assigned, and the corresponding points were summed to produce a total score. Individual scores were then combined to calculate the total score MSSI. MSSI was evaluated as *mild* for values between 0–20, *moderate* for values between 20–40, and *severe* for values higher than 40. Baseline MSSI values were obtained before treatment, follow up values after at least 1 year of ERT. Patients were classified as non-responders if MSSI values changes from *mild* (at baseline) to *moderate* or from *moderate* to *severe*.

### Methods

Genomic DNA was extracted from peripheral blood using Perfect Pure DNA Blood kit (5 Prime) and analyzed using the DMET Plus assay (Affymetrix, Santa Clara, CA), as previously described [31–35]. DMET Console version 1.1 (Affymetrix, Santa Clara, CA) was used to perform genotype calls from intensity array data using the Dynamic Genotype Boundaries algorithm. We applied a call rate less than 95% as exclusion criteria from further analysis. Association analysis was performed using DMET-Analyzer Tool software [36]. The observed genotype frequencies for each SNP were tested for HWE in both groups, responders and non-responders, using χ^2^ test. Odds ratios (ORs) and corresponding 95% confidence intervals (CIs) were calculated for 2 × 2 table using Med Calc v12.3.0. Unpaired *t*-test and Bonferroni correction were done in GraphPad Prism 7 (GraphPad Software, Inc).

All genotypes of interest were validated using pre-designed TaqMan SNP genotyping assays (Assay ID: C_519458_40, C_11941799_30, C_11941805_40 and C_9523470_10, Applied Biosystem). PCR amplification and endpoint plate read were carried out on a ViiA7^TM^ Real-Time PCR System (Applied Biosystem). All reactions were performed in duplicate in a final volume of 10 μL accordingly to the manufacturer's recommendations. Mismatched genotypes, which constituted < 0.5% of the total number of duplicate genotypes performed, were discarded.

## Abbreviations

ADME: Adsorption, distribution, metabolizing enzyme
α-GalA: α-galactosidase A
DMET: Drug metabolizing enzyme and transporters
ERT: Enzyme replacement therapy
FD: Fabry disease
Gb3: Globotriaosylceramide
4-HNE: 4-hydroxynonenal
HWE: Hardy–Weinberg equilibrium
PCR: Polymerase chain reaction (PCR)
SNP: Single nucleotide polymorphism
